# GNSS Observation Generation from Smartphone Android Location API: Performance of Existing Apps, Issues and Improvement

**DOI:** 10.3390/s23020777

**Published:** 2023-01-10

**Authors:** Farzaneh Zangenehnejad, Yang Jiang, Yang Gao

**Affiliations:** Department of Geomatics Engineering, Schulich School of Engineering, University of Calgary, Calgary, AB T2N 1N4, Canada

**Keywords:** smartphone positioning, precise point positioning (PPP), android location API, smartphone GNSS logging apps, GnssLogger app, Geo++ RINEX logger app, UofC CSV2RINEX tool

## Abstract

Precise position information available from smartphones can play an important role in developing new location-based service (LBS) applications. Starting from 2016, and after the release of Nougat version (Version 7) by Google, developers have had access to the GNSS raw measurements through the new application programming interface (API), namely android.location (API level 24). However, the new API does not provide the typical GNSS observations directly (e.g., pseudorange, carrier-phase and Doppler observations) which have to be generated by the users themselves. Although several Apps have been developed for the GNSS observations generation, various data analyses indicate quality concerns, from biases to observation inconsistency in the generated GNSS observations output from those Apps. The quality concerns would subsequently affect GNSS data processing such as cycle slip detection, code smoothing and ultimately positioning performance. In this study, we first investigate algorithms for GNSS observations generation from the android.location API output. We then evaluate the performances of two widely used Apps (Geo++RINEX logger and GnssLogger Apps), as well as our newly developed one (namely UofC CSV2RINEX tool) which converts the CSV file to a Receiver INdependent Exchange (RINEX) file. Positioning performance analysis is also provided which indicates improved positioning accuracy using our newly developed tool. Future work finding out the potential reasons for the identified misbehavior in the generated GNSS observations is recommended; it will require a joint effort with the App developers.

## 1. Introduction

Precise position information available from smartphones is of great importance when enabling many smartphone-based location-based service (LBS) applications. Since the GNSS observations, including carrier-phase, became available to users from smart devices running Android Nougat (version 7.0) in 2016, many methods and algorithms have been developed to enable precise positioning using these mass-market devices, such as analysis of GNSS smartphone observations [1,2,3,4,5], PPP smartphone positioning [6,7,8,9,10], real-time kinematic (RTK) smartphone positioning [11,12,13,14,15] and GNSS/INS integration using smartphone observations [16,17,18]. A comprehensive review of the recent advances and research done in the field of GNSS smartphone positioning, including those published up until 2021, can be found in [19,20], while some more recent contributions in 2022 are provided in the following. Bahadur [21] investigated the real-time standalone positioning accuracy employing the single-frequency code observations form the three smartphones, Xiaomi Mi8, Google Pixel 4 and Pixel 4 XL, in the kinematic mode. The study addressed two issues: (1) comparing the ultra-rapid and IGS real-time service (IGS-RTS) and (2) investigating the effect of an improved weighting model, utilizing the variance component estimation method, on the positioning accuracy. The experimental tests indicated that the use of IGS-RTS products augments had better performance compared with the ultra-rapid products. Moreover, introducing the actual stochastic characteristics of multi-GNSS observations improved the smartphone positioning performance by 11.0% on average. Li et al. [22] proposed a combined elevation angle and carrier-to-noise density ratio (C/N0) weighting method for smartphone-based GNSS PPP by normalizing the C/N0-derived variances to the scale of the elevation-angle-derived variances. The results indicated an improvement in the three-dimensional positioning accuracy by 22.7% and 24.2% in an open-sky area, and by 52.0% and 26.0% in a constrained visibility area, compared with the elevation-angle-only and C/N0-only weighting methods, respectively. Li and Cai [23] proposed a mixed single- and dual-frequency quad-constellation PPP model to improve the smartphone positioning performance by taking advantage of all available GNSS observations. The effectiveness of the proposed model has then been investigated using both static and kinematic tests. Based on the results, the mixed frequency model could effectively improve the positioning performance compared to the traditional dual-frequency PPP and the single-frequency PPP. Li et al. [24] proposed a real-time PPP algorithm for land vehicle navigation with smart devices. The smartphones were placed on the roof and the dashboard. The positioning accuracy of vehicle-roof mode was in the order of 1.0 m for the horizontal component and 1.5 m for the vertical component, while the positioning accuracy of the dashboard test were about 1.0–1.5 m in the horizontal direction and 1–2 m in the vertical direction. Li et al. [25] proposed an uncombined PPP-RTK model to achieve rapid integer ambiguity resolution (IAR) with the regional atmospheric augmentation with an external low-cost helical antenna. The results indicated that PPP rapid ambiguity resolution could be achieved using the smart devices’ GNSS raw observations with a low-cost helical antenna. The method, therefore, has the potential to provide high-precision positioning services and can be widely used in massive market applications because of the advantages of low weight, low-power consumption and portability. Xu et al. [26] investigated the performance of ionospheric total electron content (TEC) determined by GNSS dual-frequency measurements derived from the Xiaomi Mi8, as an example. In this contribution, the ionospheric observable was retrieved from the code and carrier-phase measurements using the carrier-to-code leveling technique and a new carrier-to-noise weighting strategy instead of an elevation weighting strategy. The slant TEC derived from the Xiaomi Mi8 was then compared to the slant TEC derived from a geodetic receiver as the reference. According to the results, applying smart device-level GNSS observations in ionospheric studies is feasible. Zhu et al. [27] proposed an inertial measurement unit (IMU)-aided uncombined PPP coupled mathematical model, suitable for smartphone positioning. The proposed PPP/INS-coupled model integrated the dual-frequency GNSS observations and IMU data from smartphones with C/N0-dependent stochastic model and robust Kalman filter (RKF) model to improve the positioning performance under GNSS-degraded environments. Experimental results indicated that the proposed PPP/INS method could effectively improve the smartphone positioning performance compared with the conventional smartphone PPP method. Yi et al. [28] presented a novel sensor fusion technique using PPP and the inertial sensors in smartphones, combined with a single- and dual-frequency (SFDF) optimisation scheme for smartphones. Using several vehicle experiments, a significant improvement in the final solutions has been confirmed in the case of multi-GNSS PPP/IMU integration, providing consistent horizontal positioning accuracy of <2 m RMS in real-world driving scenarios.

Among the contributions in the field of differential/relative positioning, we can also refer to the following. Bakuła et al. [29] investigated the effect of L1 and L5 frequencies on the positioning accuracy of the pseudo-range differential GNSS (DGPS) using data from two Huawei P30 Pro devices. The results showed a better positioning accuracy employing the P(L5) code compared to the P(L1) code. Li et al. [30] proposed a combined RTK/fifth generation (5G) mobile communication technology positioning model by combining global positioning system-RTK with 5G time-of-arrival observations to improve the positioning accuracy under medium and long baselines. The results indicated that good positioning results could be achieved in the case of combined RTK/5G positioning model, even while some satellites are occluded. Benvenuto et al. [31] presented a method for mitigating the multipath effect in order to improve the accuracy and robustness of GNSS RTK positioning from Android smartphones. The main idea is to weigh GNSS observations of each piece of satellite data considering the proposed parameter MDP (multipath detection parameter) and signal noise ratio (SNR) values. It assigns lower weights to the unreliable observations potentially affected by the multipath error (and vice versa). Li et al. [32] proposed a single-frequency RTK robust adaptive Kalman filtering algorithm applied to smartphone GNSS positioning. It is based on the quartile method to dynamically determine the threshold value and eliminate the gross error of observation. The effectiveness of the proposed quartile robust RTK algorithm has been validated using the simulated and real dynamic experiments. According to the results, the proposed method could significantly eliminate large gross errors, and reasonably allocate weights to different observations according to the innovation vector. As a result, the overall positioning accuracies have been improved. Liu et al. [33] proposed a method to detect and correct the non-line-of-sight (NLOS) signals, which is an important issue in urban environments. This method is based on a convolutional neural network constructed using the original observations of smartphones providing the detection accuracy of more than 95%. The detected NLOS signals were decomposed using the variational mode decomposition method to eliminate the NLOS part and improve the data quality. They then evaluated the efficiency of the proposed method in both static and dynamic modes in an urban environment using the RTK method. The results confirmed an improvement in the RTK positioning accuracy in both static and dynamic tests using the proposed method. Various researchers have also been conducting investigations into the feasibility of ambiguity resolution with a smartphone receiver. For example, Miao et al. [34] first investigated the quality of L5/E5a/B2a signals, their superiorities in IAR and precise positioning with respect to the L1/E1/B1 signals from GPS, QZSS, Galileo and Beidou−2/3 satellites. The authors then proposed a new weighting model that takes into account the variation range of C/N0, providing a better weighting model than the traditional weighting model, thus improving the positioning accuracy. The results indicated that the L5/E5a/B2a signals could generally obtain higher IAR fix-rate and positioning accuracies than the L1/E1/B1 signals. Yong et al. [35] compared the best integer equivariant (BIE) estimator to the integer least squares (ILS) and float contenders using GNSS data collected by Google Pixel 4 smartphones for short-baseline RTK positioning. The results indicated that the BIE estimator will always give a better RTK positioning performance than that of the ILS and float solutions while using both single- and dual-frequency smartphone measurements for the combination of GPS + Galileo + QZSS + BDS. Li et al. [36] investigated the effect factors for integer property of phase ambiguities, data quality, IAR efficiency and positioning accuracy for the smartphone. The results indicated that the smartphone brands, operating systems and smartphone attitudes would affect the integer property of phase ambiguities and data quality. The kinematic positioning results showed the meter-level accuracy with an embedded antenna, and the centimeter to decimeter-level accuracy with the external antenna. Finally, we should note that although the results of current studies are promising, we still need more effort to achieve satisfying accuracy for many location-based services.

In 2021 and 2022, the Android GPS team of Google hosted two Google smartphone decimeter challenges (GSDC), where various smartphone GNSS datasets of real vehicular applications were used to determine smartphone positioning accuracies [37]. It was revealed that meter-level accuracy is generally achieved by the leading participants, which is still not enough to enable smartphone precise positioning. Several challenges must be taken into account in order to further improve smartphone positioning such as: carefully analyzing the smartphone GNSS observations, investigating the environment effect and smartphone holding modes and improving positioning algorithm and implementation.

It is obvious that the quality of the GNSS observations plays an important role in the final positioning performance. Currently the GNSS pseudorange, carrier-phase and Doppler observations are not directly accessible from the API 24 implemented in Android 7 or higher. They have to be generated by smartphone users from the GNSS raw information accessible through Google’s application programming interface (API), namely android.location (API level 24). Various Apps for smartphone GNSS data logging and GNSS observations generation have been developed and two of them are widely used as described in the following. In 2016, Google released an open source application, namely GnssLogger App, which logs the raw measurements of the GnssClock and GnssMeasurement classes from the android.location API. This information can then be used to generate the GPS time, pseudorange, carrier-phase and Doppler observations, which, however, must be done by the users themselves. Later In 2017, the Geo++ GmbH Company released an open-source application, namely Geo++ RINEX Logger App, capable of providing the GNSS pseudorange, carrier-phase and Doppler observations in the Receiver INdependent Exchange (RINEX) format [38]. The GnssLogger App in its updated version (v3.0.0.1) can now provide not only the android.location API raw GNSS measurements in CSV format, but also GNSS observations in the RINEX format. Generating the carrier-phase and Doppler observations is straightforward (refer to Equations (3) and (4) in Section 2). However, generating the pseudorange observations might be challenging. Some further information can be found in Section 2.

Although GnssLogger and GEO++ RINEX Logger are widely used, the quality of GNSS observations output from them was found inconsistent in different aspects. In a previous work based on dataset from the Xiaomi Mi8, Google Pixel 5 and Samsung S20 smartphones, the first-order differences of pseudorange and carrier-phase observations were found not following the same trend of the Doppler observations for all three smartphones. The results also showed that the Doppler observations from the Xiaomi Mi8 and Samsung S20 smart devices were biased with respect to the pseudorange and carrier-phase observations [39]. The data logging in the RINEX format (i.e., generating the typical GNSS observations from the Android location API) was likely the cause of such observations’ misbehavior. This is due to the fact that each logging App implements its own GNSS observation conversion algorithm and uses different parameter settings, thresholds and float computing accuracies. The quality concerns in the generated GNSS observations would affect GNSS data processing such as cycle slip detection, code smoothing and ultimately positioning performance. Since the data logging and GNSS observations generation are a critical part of smartphone positioning algorithm development, they should be carefully evaluated for the purpose of precise position determination. A comparison between different Apps should therefore be made to assess their consistency.

The aim of this paper is to investigate the quality of smartphone GNSS observations in RINEX format from existing smartphone GNSS logging Apps and improvement in smartphone GNSS observation generation with a focus on the following aspects:

- To provide a performance evaluation of RINEX outputs from two widely used smartphone GNSS data loggers, namely the GnssLogger App, and the Geo++ RINEX App, and also compare to our newly developed software (UofC CSV2RINEX tool). It gives the reader a great insight into the potential issues in the GNSS observations such as their inconsistency and bias issues in the smartphone pseudorange, carrier-phase and Doppler observations;
- To introduce our newly developed software (UofC CSV2RINEX tool) available at https://github.com/FarzanehZangeneh/csv2rinex, which provides improved performance. Such a tool is of value to researchers and engineers who are developing precise positioning algorithms and products with smartphones GNSS observations;
- To investigate the positioning performance of the three RINEX files in the post-processed mode using a real kinematic dataset.

The paper is organized as follows. How to convert the Android raw location-related measurements to the typical GNSS observations (e.g., pseudorange, carrier-phase and Doppler) is first explained in the next section. In the subsequent section, the employed mathematical model, which is the uncombined precise point positioning (UPPP) model, is described in detail. In the numerical results section, the quality of generated GNSS observations from different smart devices and using different loggers is assessed. In this section, the inconsistency between the pseudorange, carrier-phase and Doppler measurements reported also in Zangenehnejad et al. [39] is thoroughly investigated. The presence of some carrier-phase observations without changes over time and its possible reason will also be addressed in this part. Finally, the positioning performance of the three RINEX files (RINEX outputs from GnssLogger, Geo++ RINEX logger and UofC CSV2RINEX convertor) is investigated using the GNSS observations from the Xiaomi Mi8 in kinematic mode. We then draw some conclusions in the last section.

## 2. Access to Android Raw GNSS Measurements and GNSS Observation Generation

Since releasing the Nougat version of the Android system (Version 7) in 2016, the users have access to the raw GNSS measurements through the new location API consisting of two classes, GNSSMeasurement class and GNSSClock class. However, the users still need to extract the typical GNSS observations, such as pseudorange, carrier-phase and Doppler observations, from the raw data logged in the two classes. A list of raw measurements of Android 7 Location API in GNSSClock and GNSSMeasurement classes can be found in [20,40]. How to convert the raw measurements logged through the android.location API to the GNSS observations (i.e., pseudorange, carrier-phase and Doppler observations) has been provided in details in the white paper published by the European GNSS Agency’s (GSA) [40]. Table 1 gives a list of available GNSS logger Android applications and their output formats.

A brief explanation about how to generate the pseudorange, carrier-phase and Doppler observations is provided in continue.

### 2.1. Pseudorange Observation Generation

The pseudorange observation is the travelling time of the signal to propagate from the satellite to the receiver (here smartphone). It is of the form [24]:

(1) $$P=\left(t_{Rx}-t_{Tx}\right)\times 10^{-9}\times c,$$

where P is the pseudorange observation in meter, $t_{Rx}$ is the received time (measurement time) in nanosecond, $t_{Tx}=ReceivedSvTimeNanos\;\left[\mathrm{ns}\right]$ is the received GNSS satellite time at the measurement time in nanosecond reported in the CSV file (one of the variables in the GNSSMeasurement class) and $c=299792458.0\;\left[\text{m/s}\right]$ is the speed of light. The measurement time $t_{Rx}$ in GNSS time system in nanosecond is as follows:

(2) $$t_{Rx\;GNSS}=TimeNanos+TimeOffsetNanos-\left(FullBiasNanos\left(1\right)+BiasNanos\left(1\right)\right),$$

where TimeNanos is the GNSS receiver’s internal hardware clock value, TimeOffsetNanos is the time offset at which the measurement was taken, FullBiasNanos is the difference between TimeNanos inside the GPS receiver and the true GPS time since 6 January 1980 and BiasNanos is the clock’s sub-nanosecond bias. All of these variables can be found either in the GNSSMeasurement class or in the GNSSClock class. They all are reported in nanosecond. It should be noted that only the first value of FullBiasNanos and BiasNanos must be used to compute all the received times (i.e., $FullBiasNanos\left(1\right)$ and $BiasNanos\left(1\right)$) as long as there is no discontinuity in the internal received time. $t_{Rx\;GNSS}$ and $t_{Tx}$ must also be in the same time system for all GNSS systems which is not the case here as $t_{Rx\;GNSS}$ is in the GNSS reference system while $t_{Tx}$ is given for each GNSS system. Therefore, one must convert to other one (i.e., same GNSS time system). How to do this can be found in [20,40].

### 2.2. Carrier-Phase Observation Generation

The carrier-phase observation in cycle can be obtained as:

(3) φ=AccumulatedDeltaRangeMeters/λ,

where AccumulatedDeltaRangeMeters is the accumulated delta range (ADR) since the last channel reset which is one of the variables from GNSSMeasurement class within the Android API package “location”. λ also denotes the signal’s wavelength. It should also be noted that it is better to use only valid measurements for the carrier-phase observation calculation. Validity of the carrier measurements can be checked using the AccumulatedDeltaRangeState variable.

### 2.3. Doppler Observation Generation

The Doppler shift causing from the satellite movement can be obtained as follows:

(4) dopplershift=−PseudorangeRatemetersperSecond/λ,

where PseudorangeRatemetersperSecond is the pseudorange rate at the timestamp in m/s and can be found as one of the variables in GNSSMeasurement class.

Computing the carrier-phase and Doppler observations are straightforward and we do not face any numerical problems while converting them. However, we might have some numerical issues/errors while generating the pseudorange observations. This is due to the fact that each logging App implements its own GNSS observation conversion algorithm and uses different parameter settings, thresholds and float computing accuracies. Such an issue will affect the quality of the generated observations saved into the RINEX file. In this contribution, we have also developed our in-house convertor in C++, namely UofC CSV2RINEX, to convert a CSV file into a RINEX file. In Section 4, three RINEX files coming from GnssLogger App, Geo++ RINEX logger and our converted RINEX using UofC CSV2RINEX are thoroughly investigated and compared from different aspects.

## 3. Precise Positioning Using Uncombined PPP (UPPP) Algorithm

With GNSS observations of pseudorange, carrier-phase and Doppler, the uncombined PPP (UPPP) model can be employed for precise positioning. The undifferenced GNSS pseudorange and carrier-phase observations for the satellite *s* and the receiver *r* on frequency *j* are as follows [41]:

(5) $$\begin{array}{l} E\left(\Phi_{r,j}^{s}\right)=\rho_{r}^{s}+T_{r}^{s}+cdt_{r}-cdt^{s}-\gamma_{j}I_{r,1}^{s}+\lambda_{j}N_{r,j}^{s}+B_{r,j}-B_{j}^{s}\\ E\left(P_{r,j}^{s}\right)=\rho_{r}^{s}+T_{r}^{s}+cdt_{r}-cdt^{s}+\gamma_{j}I_{r,1}^{s}+b_{r,j}-b_{j}^{s} \end{array},$$

where *E* is the mathematical expectation operator, $P_{j}$ and $\Phi_{j}=\lambda_{j}\varphi_{j}$ denote the pseudorange and carrier-phase observations on the frequency j in meters, ρ is the geometric range between satellite and receiver as a function of the satellite and the receiver coordinates, T is the tropospheric delay (m) which can be spilt into dry and wet parts, c is the vacuum speed of light (m/s), $dt_{r}$ and $dt^{s}$ are the receiver and satellite clock errors (s), respectively, $I_{r,1}^{s}$ is the first-order slant ionospheric delay on frequency L1 (m), γj=f12fj2 is the frequency-dependent multiplier factor (in the case of L1 frequency$\;\gamma_{j}=1$), $f_{f}$ is the corresponding frequency, $\lambda_{j}$ is the corresponding carrier-phase wavelength (m), $N_{r,j}^{s}$ denotes the integer carrier-phase ambiguity term in cycle, $b_{r,j}$ and $B_{r,j}$ denote the frequency-dependent receiver pseudorange and carrier-phase hardware delays (biases), respectively, and $b_{f}^{s}$ and $B_{f}^{s}$ are the frequency-dependent satellite pseudorange and carrier-phase hardware delays (biases), respectively.

The precise satellite clock errors provided by International GNSS Service (IGS) are based on the ionosphere-free (*IF*) linear combination of code observations on L1 and L2 frequencies, i.e., P1 and P2, as follows [42]:

(6) $$dt^{s,IF}=cdt^{s}+b_{IF\left(1,2\right)}^{s},$$

where $b_{IF\left(1,2\right)}^{s}=\alpha_{IF}^{L1,L2}b_{1}^{s}+\beta_{IF}^{L1,L2}b_{2}^{s}\;$is the satellite ionosphere-free code bias in which $b_{1}^{s}$ and $b_{2}^{s}$ are the satellite pseudorange hardware delays for P1 and P2, respectively. The coefficients $\alpha_{IF}^{L1,L2}\;$and $\beta_{IF}^{L1,L2}$ are also of the following form:

(7) αIFL1,L2=f12f12−f22 and βIFL1,L2=−f22f12−f22,

The uncombined PPP model for L1 and L5 frequencies can then be rewritten as follows:

(8) $$\begin{array}{l} E\left(P_{r,1}^{s}\right)=\rho_{r}^{s}+T_{r}^{s}+\left(cdt_{r}+b_{r,1}\right)-cdt^{s,IF}+I_{r,1}^{s}+\left(b_{IF\left(1,2\right)}^{s}-b_{1}^{s}\right)\\ E\left(\Phi_{r,1}^{s}\right)=\rho_{r}^{s}+T_{r}^{s}+\left(cdt_{r}+b_{r,1}\right)-cdt^{s,IF}-I_{r,1}^{s}+[\lambda_{1}N_{r,1}^{s}-b_{r,1}+\;B_{r,1}-B_{1}^{s}+b_{IF\left(1,2\right)}^{s}]\;\\ E\left(P_{r,3}^{s}\right)=\rho_{r}^{s}+T_{r}^{s}+\left(cdt_{r}+b_{r,1}\right)-cdt^{s,IF}+\gamma_{3}I_{r,1}^{s}+b_{r,3}-b_{r,1}+\left(b_{IF\left(1,2\right)}^{s}-b_{3}^{s}\right)\\ E\left(\Phi_{r,3}^{s}\right)=\rho_{r}^{s}+T_{r}^{s}+\left(cdt_{r}+b_{r,1}\right)-cdt^{s,IF}-\gamma_{3}I_{r,1}^{s}+[\lambda_{3}N_{r,3}^{s}+B_{r,3}-B_{3}^{s}+b_{IF\left(1,2\right)}^{s}-b_{r,1}] \end{array},$$

By introducing ${\tilde{cdt}}_{r}=cdt_{r}+b_{r,1}$,$\;\lambda_{1}{\tilde{N}}_{r,1}^{s}=\lambda_{1}N_{r,1}^{s}+\;B_{r,1}-B_{1}^{s}+b_{IF\left(1,2\right)}^{s}-b_{r,1}$ and $\lambda_{3}{\tilde{N}}_{r,3}^{s}=\lambda_{3}N_{r,3}^{s}+B_{r,3}-B_{3}^{s}+b_{IF\left(1,2\right)}^{s}-b_{r,1}$, have:

(9) $$\left\{\begin{array}{l} E\left(P_{r,1}^{s}-\frac{1}{\gamma_{2}-1}DCB_{1,2}^{s}\right)=\rho_{r}^{s}+T_{r}^{s}+{\tilde{cdt}}_{r}-cdt^{s,IF}+I_{r,1}^{s}\\ E\left(\Phi_{r,1}^{s}\right)=\rho_{r}^{s}+T_{r}^{s}+{\tilde{cdt}}_{r}-cdt^{s,IF}-I_{r,1}^{s}+\lambda_{1}{\tilde{N}}_{r,1}^{s}\\ E\left(P_{r,3}^{s}-\frac{\gamma_{2}}{\gamma_{2}-1}DCB_{1,2}^{s}+\frac{1}{\gamma_{2}-1}DCB_{2,3}^{s}\right)=\rho_{r}^{s}+T_{r}^{s}+{\tilde{cdt}}_{r}-cdt^{s,IF}+\gamma_{3}I_{r,1}^{s}-DCB_{1,3}^{r}\\ E\left(\Phi_{r,3}^{s}\right)=\rho_{r}^{s}+T_{r}^{s}+{\tilde{cdt}}_{r}-cdt^{s,IF}-\gamma_{3}I_{r,1}^{s}+\lambda_{3}{\tilde{N}}_{r,3}^{s}\; \end{array}\right.,$$

where $DCB_{1,3}^{r}=b_{r,1}-b_{r,3}$, $b_{IF\left(1,2\right)}^{s}-b_{1}^{s}=\frac{1}{\gamma_{2}-1}DCB_{1,2}^{s}$, $b_{IF\left(1,2\right)}^{s}-b_{3}^{s}=\frac{\gamma_{2}}{\gamma_{2}-1}DCB_{1,2}^{s}-\frac{1}{\gamma_{2}-1}DCB_{2,3}^{s}$ with $DCB_{1,2}^{s}=b_{1}^{s}-b_{2}^{s}$, $DCB_{2,3}^{s}=b_{2}^{s}-b_{3}^{s}$ which are the satellite differential code biases (DCB) available from the IGS. The unknowns here are the receiver position, the receiver clock error ${\tilde{cdt}}_{r}$, the real-valued carrier-phase ambiguity terms $\lambda_{1}{\tilde{N}}_{r,1}^{s}$ and $\lambda_{3}{\tilde{N}}_{r,3}^{s}$, the slant ionospheric delay $I_{r,1}^{s}$, the tropospheric delay and $DCB_{1,3}^{r}$. The ionospheric delay can be also modeled by the global ionospheric maps (GIM) or the empirical models, i.e., ionosphere-corrected.

## 4. Quality Analysis of GNSS Observations from Different Logging Apps and Improvement

This section consists of two parts. First, the quality of GNSS measurements saved into the RINEX files obtained from the two widely used logging Apps, namely GnssLogger and Geo++ RINEX logger, is assessed from different aspects. In this section, we also assess our newly developed in-house software (UofC CSV2RINEX) for converting a CSV file into a RINEX file which provides improved GNSS observations. Second, the positioning performance of the three RINEX files is investigated in kinematic mode.

### 4.1. Analysis of GNSS Observations from Different Logging Apps

In this section, we first thoroughly investigate the inconsistency between the pseudorange, carrier-phase and Doppler observations in Section 4.1.1. Such misbehavior has been recently reported in [39]. Another issue which will be covered in Section 4.1.2 is the presence of some carrier-phase observations without changes over time. The results of these two subsections clearly indicate the importance of analyzing GNSS logger outputs before using them.

#### 4.1.1. Inconsistency between Pseudorange, Carrier-Phase and Doppler Observations

GnssLogger app and Geo++ RINEX both are capable of providing GNSS observables in RINEX format. Having RINEX format available allows the users to post-process the logged data, improving the accuracy. However, different logging apps have different performance which affects the positioning results as well. In this section, we first investigate outputs of the three RINEX files, (1) RINEX file saved by GNSSLogger App, (2) RINEX file logged by Geo++ RINEX Logger App and (3) RINEX file generated by our convertor toolbox which converts the CSV file to the RINEX format.

To this end, the Xiaomi Mi8, Samsung S20 and Google Pixel 5 smartphones were put on the top of the geodetic pillars with known coordinates on the rooftop of the Civil Engineering building, University of Calgary, Calgary, Canada. The first two devices used the Broadcom chipset, while the last one used the Qualcomm chipset. All three devices were dual-frequency smartphones supporting L5/E5a frequencies for GPS and Galileo, respectively. The dataset was collected on 23 November 2022 with a sampling interval of 1 sec for about 1:30 h. For further investigation and presenting results, GPS PRN 01, Galileo PRN 31 and GLONASS PRN 17 were selected. The reason for selecting these PRNs is their better availability and continuity during the observation period. We should also mention that the same results were observed for other PRNs. To have a better view, the first 900 epochs (15 min) were used for plotting the figures. Table 2 also provides a brief summary of the experiment.

**Figure 1:**
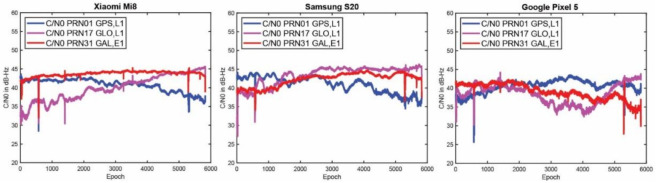
C/N0 measurements for selected PRNs and three smartphone (Xiaomi Mi8, Google Pixel 5 and Samsung S20).

Figure 1 provides the C/N0 measurements of the selected PRNs on the L1 frequency for the Xiaomi Mi8, Google Pixel 5 and Samsung S20, from left to right, respectively. The plot reveals that the three smartphones did not have similar performances in terms of their C/N0 records, even though the data have been collected in the same environment at the same time. As can be seen, the C/N0 records of the Xiaomi Mi8 and Samsung S20 are smoother than the ones recorded by the Google Pixel 5.

To investigate the quality of GNSS observations from the three RINEX files, different indicators are selected and reported in Table 3. They are as follows:

- First-order differentiation of pseudorange and carrier-phase versus Doppler observations: The first-order differentiation of GNSS pseudorange and carrier-phase observations are obtained by calculating differences between adjacent elements of GNSS pseudorange and carrier-phase observations divided by the sampling interval (i.e., $diff\left(P_{r,j}^{s}\right)/T$ and $diff\left(\Phi_{r,j}^{s}\right)/T$ where T is the sampling interval which is 1 s here). They then compare to the Doppler observations ($-\lambda_{j}D_{r,j}^{s}$). The first-order differences of pseudorange and carrier-phase observations have to follow the same trend of the Doppler observations in theory;
- Geometry-free combination (Code-minus-phase: CMP): It cancel the geometric part of the measurement (i.e., geometric range, receiver and satellite clock and tropospheric delay), leaving ambiguity, ionosphere term, multipath and noise. This combination can also be used to detect cycle-slips in the carrier-phase observations as a cycle-slip appears as a jump in the CMP plot;
- Carrier-phase predicted error: The predicted carrier-phase in cycle can be obtained using the discrete Doppler measurements as ${\hat{\varphi }}_{r,j}^{s}\left(k+1\right)=\varphi_{r,j}^{s}\left(k\right)+\frac{D_{r,j}^{s}\left(k+1\right)+D_{r,j}^{s}\left(k\right)}{2}T$. The carrier-phase predicted error is then estimated as ${\hat{\Phi }}_{r,j}^{s}-\Phi_{r,j}^{s}=\lambda_{j}{\hat{\varphi }}_{r,j}^{s}-\lambda_{j}\varphi_{r,j}^{s}$ in meters.

**Table 3:** Different indictors used to analyze raw GNSS observations.

Indicator	Formula
First-order differentiation of pseudorange and phase versus Doppler observations	$\left\{\begin{array}{c} \mathrm{diff}\left(P_{r,j}^{s}\right)/T\;\\ \mathrm{diff}\left(\Phi_{r,j}^{s}\right)/T\\ -\lambda_{j}D_{r,j}^{s} \end{array}\right.$
Geometry-free (Code minus phase: CMP)	$P_{r,j}^{s}-\Phi_{r,j}^{s}$
Carrier-phase predicted error	${\hat{\Phi }}_{r,j}^{s}-\Phi_{r,j}^{s}$

**Figure 2:**
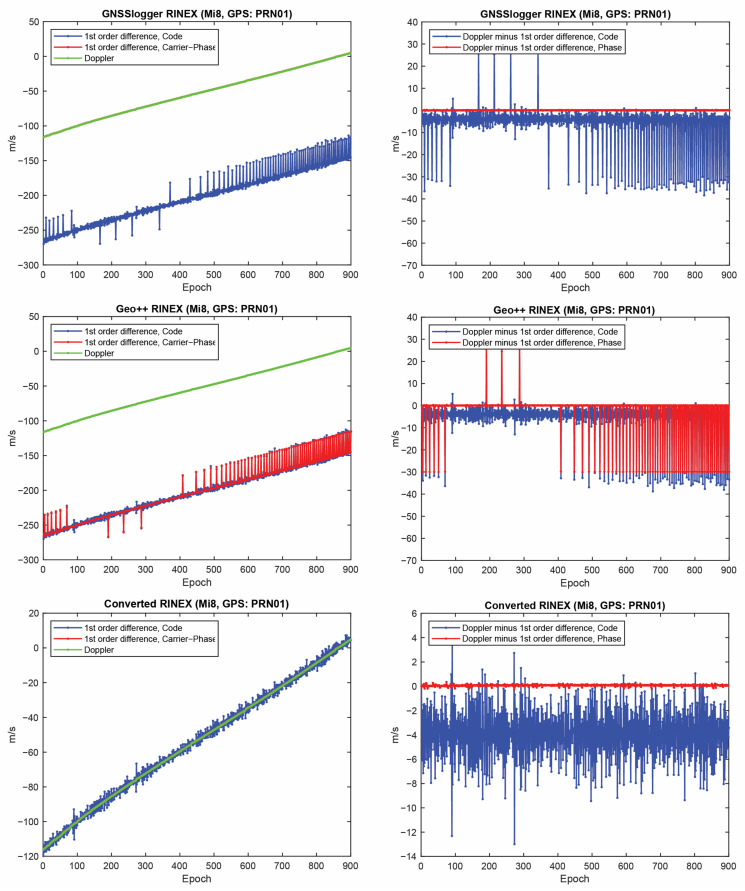
First-order differentiation of GPS pseudorange and carrier-phase observations as well as Doppler observations on L1 frequency for PRN 01 (Xiaomi Mi8).

**Figure 3:**
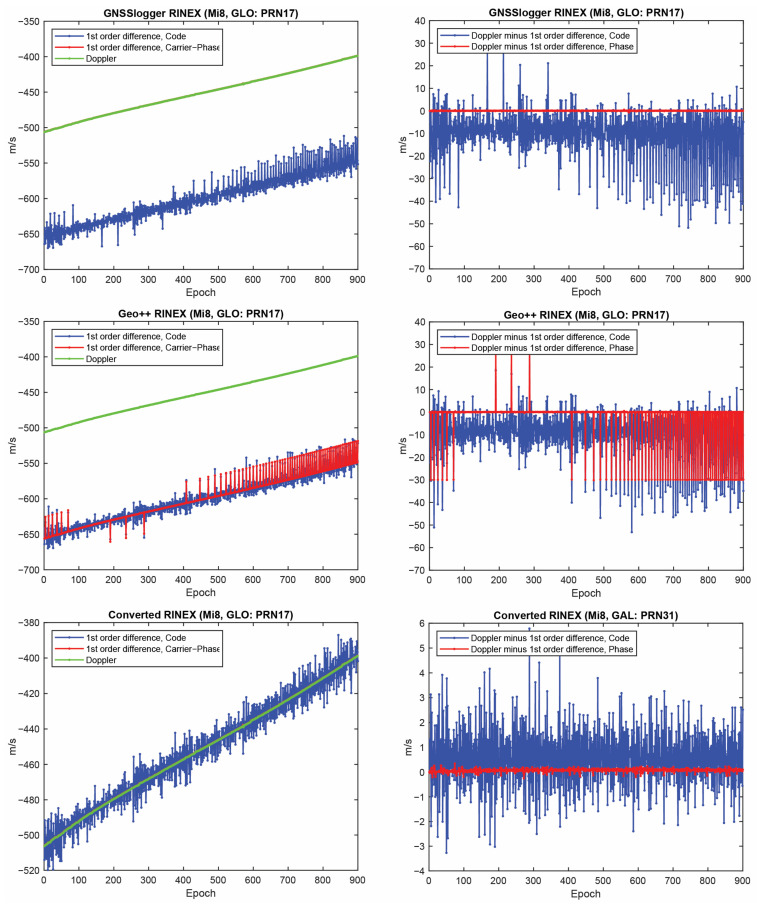
First-order differentiation of GLONASS pseudorange and carrier-phase observations as well as Doppler observations on L1 frequency for PRN 17 (Xiaomi Mi8).

**Figure 4:**
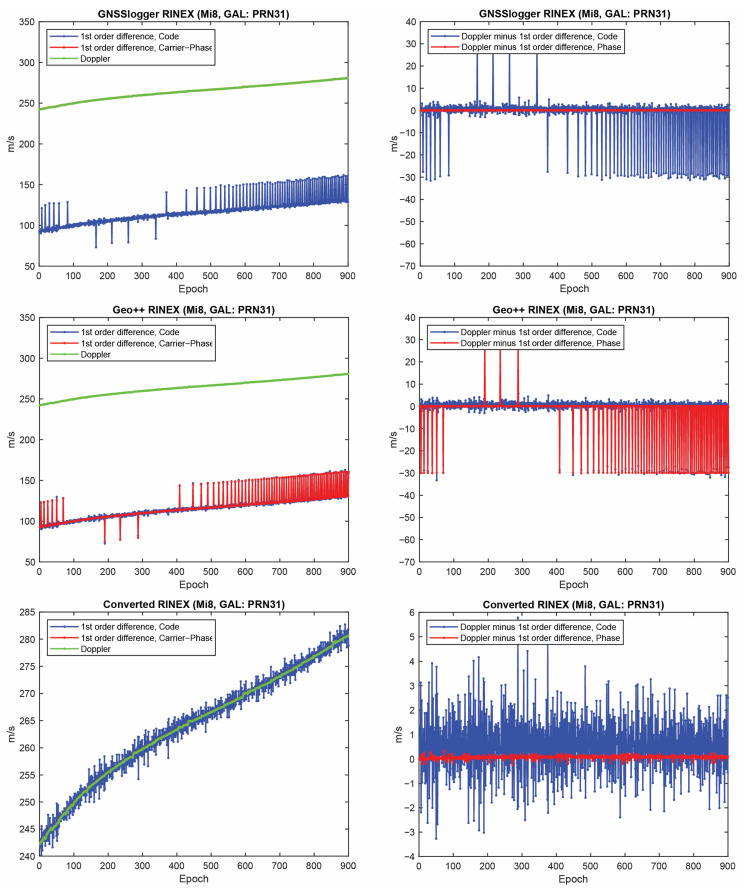
First-order differentiation of Galileo pseudorange and carrier-phase observations as well as Doppler observations on L1 frequency for PRN 31 (Xiaomi Mi8).

Let us start with the first indicator. Figure 2 shows the first-order differentiation of GPS pseudorange and carrier-phase observations, as well as the Doppler observations for PRN 01 on the L1 frequency from the three RINEX files for the Xiaomi Mi8. To have a better view, the difference between Doppler observations and the first-order differentiation of the pseudorange (i.e., $-\lambda_{j}D_{r,j}^{s}-diff\left(P_{r,j}^{s}\right)/T$) and the difference between Doppler observations and the first-order differentiation of the carrier-phase observations, (i.e., $-\lambda_{j}D_{r,j}^{s}-diff\left(\Phi_{r,j}^{s}\right)/T$) are also depicted in the right panel of this figure. In some graphs, the red line cannot be seen at this zoom setting since it is under the green one (Doppler). A few observations can be highlighted from the Figure 1. The Doppler observations of the three RINEX files are the same. As mentioned, the Doppler shift was obtained as dpplershift=−PseudorangeRatemetersperSecond/λ (see Equation (4)), showing that generating the Doppler observation was straightforward and without any complication. (2) Shown in the top panel of Figure 2 is related to the GnssLogger RINEX file. As observed, there was an offset between the Doppler and the pseudorange observations. Such an offset could have been caused during the pseudorange generation from the raw measurements in the Android API “location”-related classes. An offset was probably applied to the pseudorange observations. Applying such an offset would not be affected the solution as it could be lumped into the receiver clock bias and the real-valued ambiguities. (3) Shown in the middle panel of Figure 2 is related to the Geo++ RINEX logger output. Similar offset could be observed here not only for the pseudorange, but also for the carrier-phase observations. The carrier-phase observations followed the pseudorange observations behavior in terms of the anomalies, spikes and jumps. This shows that what happened to the pseudorange observations during their generation procedure also happened to the carrier-phase observations. (4) Shown in the bottom panel of Figure 2 is related to the converted RINEX file from our developed convertor following the equations in [40]. Unlike the other two RINEX files, there was no offset between the pseudorange and carrier-phase and Doppler observations. The first-order differences of pseudorange and carrier-phase observations also followed the same trend of the Doppler observations. (5) The Doppler observations can be employed for cycle slip detection and/or code smoothing. Considering the possible biases and anomalies in the data, the Doppler observations have to be carefully analyzed before use. The other two indicators, the CMP combination and the carrier-phase predicted error, are then utilized to further investigate the effect of possible biases and anomalies in the data. Before that, the same plots for the GLONASS PRN 17 and Galileo PRN 31 on the first frequency are shown in Figure 3 and Figure 4, respectively. Shown in the top, middle and bottom panels of Figure 3 and Figure 4 are related to the GnssLogger RINEX file, the Geo++ RINEX logger output and the converted RINEX file from our developed convertor, respectively. The same conclusions hold for the GLONASS and Galileo constellations. Therefore, we only present the GPS results in continue.

**Figure 5:**
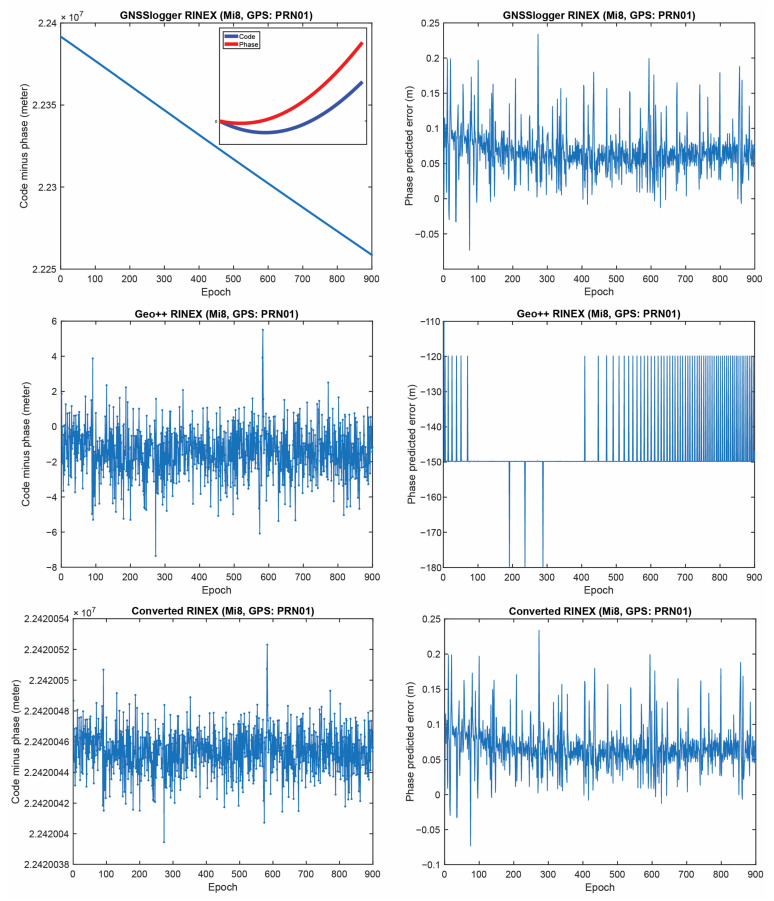
CMP (**left**) and carrier-phase predicted error (**right**) for GPS PRNs 01 (Xiaomi Mi8).

Figure 5 represents the CMP and the carrier-phase predicted error for GPS PRNs 01 from the three RINEX files for the Xiaomi Mi8 on the L1 frequency. The main purpose of this plot is to evaluate how the possible anomalies/jumps or offsets affected the CMP and the carrier-phase predicted error. The top, middle and bottom panels of Figure 5 include the CMP plot computed by using the GnssLogger RINEX, the Geo++ RINEX logger output and the converted RINEX, respectively. There are two important points about this figure that needed to be expressed. (1) First, let us look at the plot of the CMP values obtained from the GnssLogger RINEX (the top panel). The CMP does not include the geometric part while it includes the carrier-phase ambiguity, twice the ionospheric error, pseudorange and carrier-phase noise and multipath. Therefore, such behavior is not expected from the CMP values for the GnssLogger RINEX file, as it must be a constant value with a reasonable noise level as long as there is no cycle-slip in the data. It shows that the pseudorange and carrier-phase observations are not consistent (i.e., they are divergent, see the smaller panel in Figure 5 (top-left) in which the pseudorange and carrier-phase observations shifted to zero to have a better view). This indicates that the CMP combination cannot be employed to detect the possible cycle-slips in this case. We should note that this slop is the same for all PRNs. Therefore, in the case of using single-difference between satellites or double-difference observations, this issue would not affect the positioning results as it is removed through the differencing procedure. (2) As can also be observed in this figure, the carrier-phase predicted error obtained from the Geo++ RINEX logger, depicted in the middle panel, does not have an expected behavior for any reason. This also indicates that the Doppler observations cannot be employed here to detect the possible cycle-slips in the data. It is not clear to us why these plots are like that, as these Apps have not disclosed their internal algorithms.

Finally, Table 4 provides a summary of consistency or inconsistency between different GNSS observations in the three RINEX files. The highlighted cells in this table indicate that the CMP combination cannot be implemented in the cycle-slip detection procedure while using the RINEX by GNSSLogger App. In addition, the Doppler observations cannot be employed for the cycle-slip detection while using Geo++ RINEX logger output.

**Figure 6:**
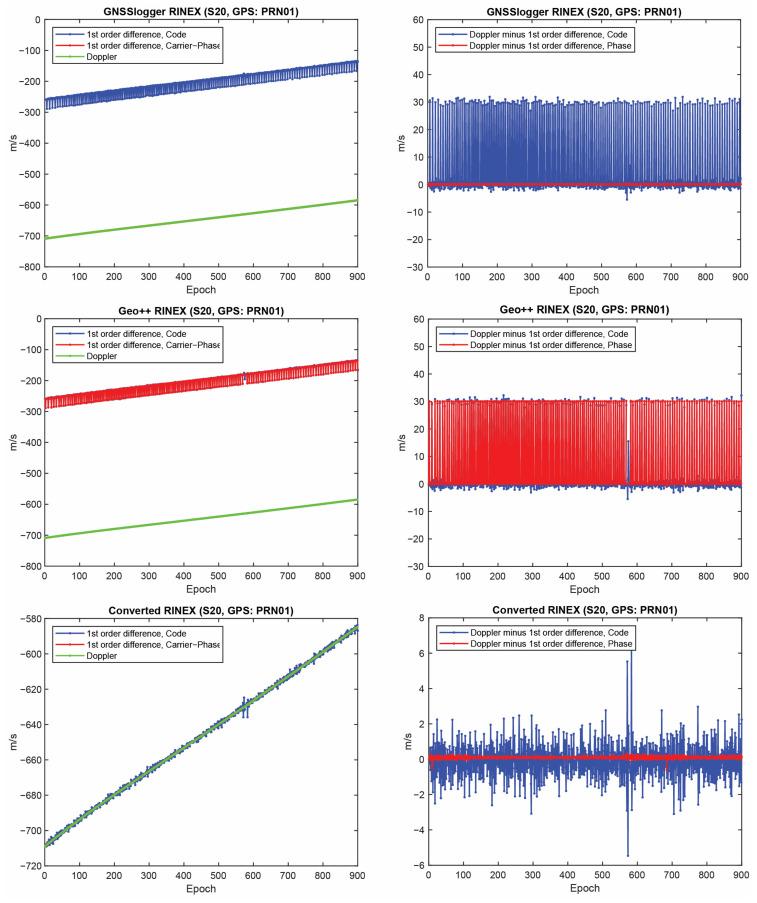
First-order differentiation of GPS pseudorange and carrier-phase observations as well as Doppler observations on L1 frequency for PRN 01 (Samsung S20).

**Figure 7:**
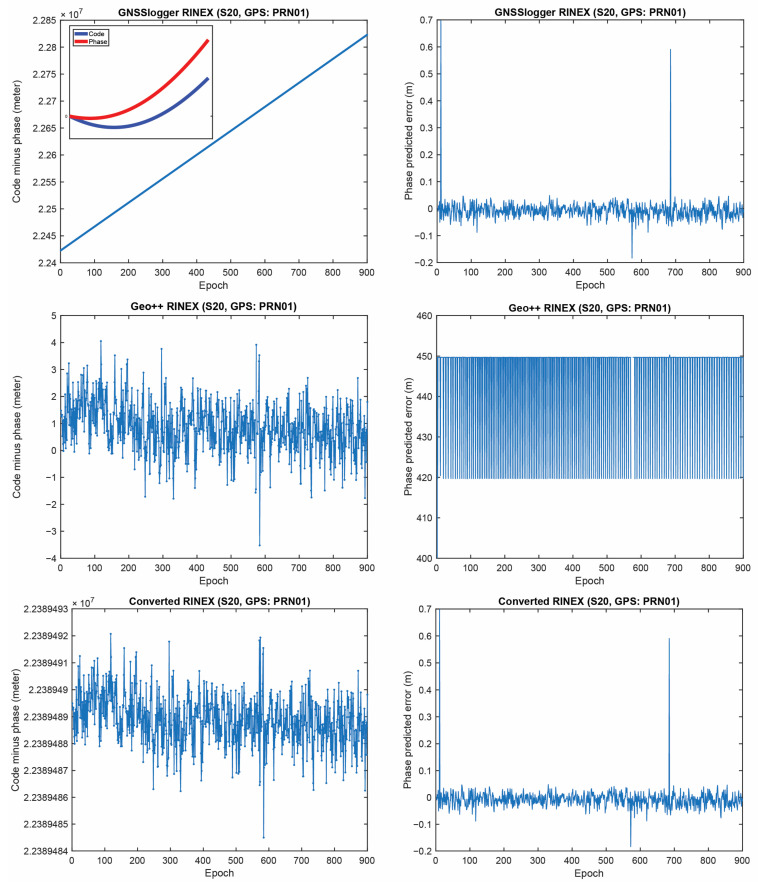
CMP carrier-phase predicted error for GPS PRNs 01 (Samsung S20).

**Figure 8:**
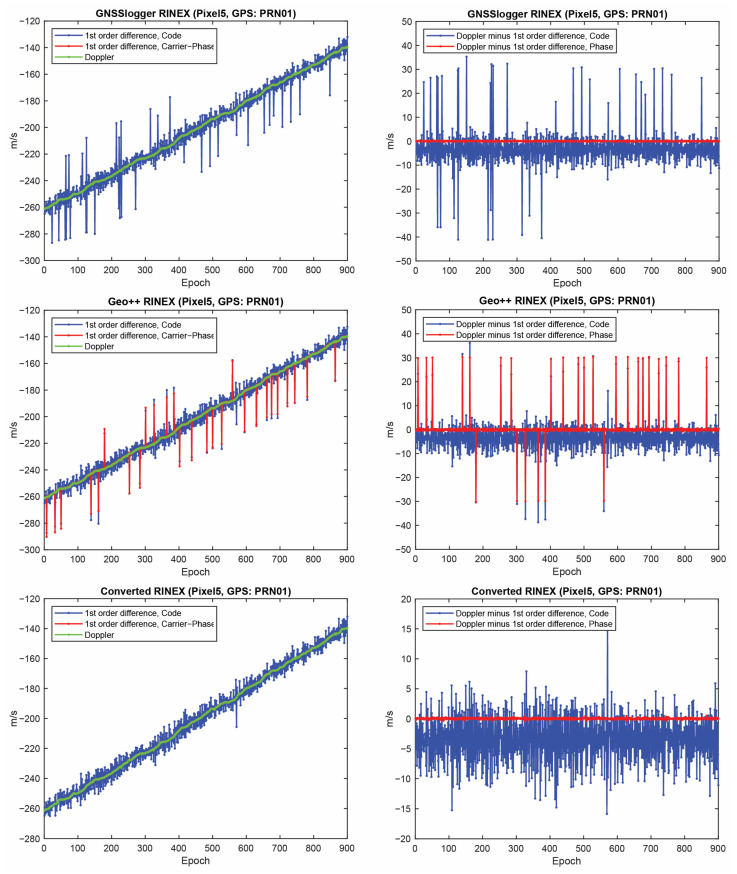
First-order differentiation of GPS pseudorange and carrier-phase observations as well as Doppler observations on L1 frequency for PRN 01 (Google Pixel 5).

**Figure 9:**
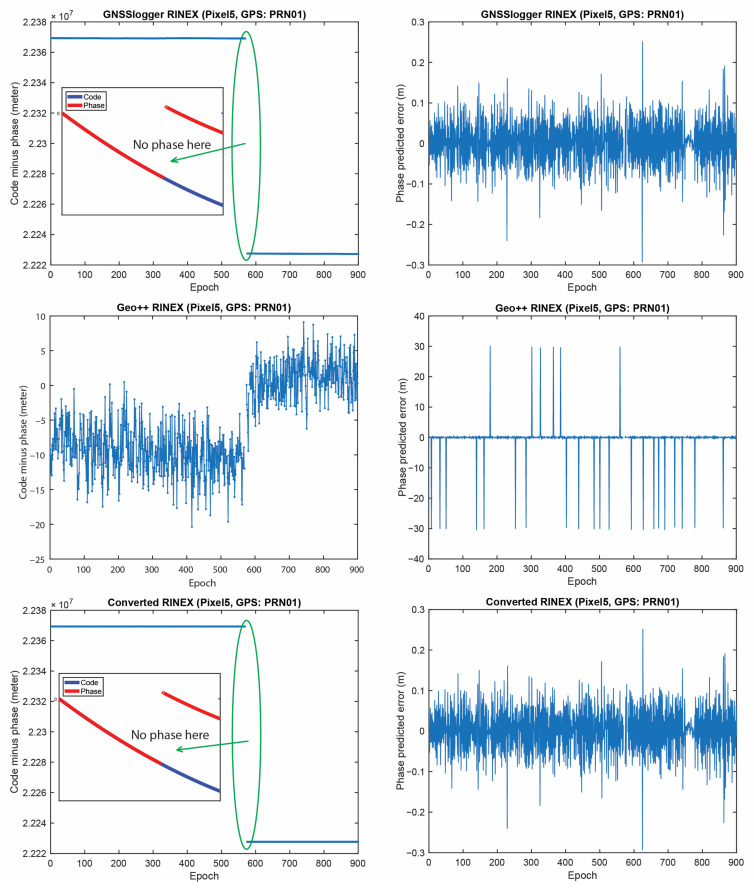
CMP and carrier-phase predicted error for GPS PRNs 01 (Google Pixel 5).

Finally, the same plots for the Samsung S20 and the Google Pixel 5 are given in Figure 6, Figure 7, Figure 8 and Figure 9. Due to the page limitation, the plots are only provided for the GPS PRN 01. They support the similar conclusion as before.

#### 4.1.2. Carrier-Phase Observations with No Change over Time

**Figure 10:**
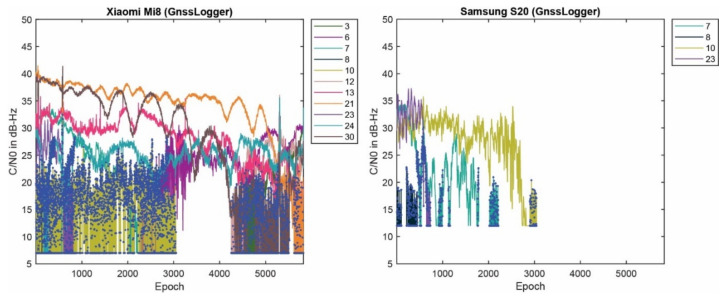
C/N0 records of GPS PRNs with problem in carrier-phase observations logs for Xiaomi Mi8 and Samsung S20 (Blue dots: epochs of carrier-phase observations with no change over time).

Another problem was also observed in the RINEX files saved by the GnssLogger App; which is the existence of some carrier-phase observations with no changes over time. Such a phenomenon was observed for the Xiaomi Mi8 and Samsung S20, while this was not the case for the Google Pixel 5 dataset. Figure 10 provides the C/N0 records for the mentioned GPS PRNs along with the epochs in which the carrier-phase observations have no changes over time for the Xiaomi Mi8 and Samsung S20, depicted in the left and right panels, respectively. They are shown with the blue dots. The number of such satellites was more for the Xiaomi Mi8 compared to the Samsung S20. Those PRNs mostly belonged to the lower C/N0 values. A C/N0 mask is usually set to 15–25 dB-Hz, however, there are still some of those epochs with the C/N0 larger than the threshold. It therefore needs further attention than just masking the lower C/N0. As given in Equation (3), the carrier-phase observation can be obtained from AccumulatedDeltaRangeMeters variable (abbreviated as ADR) from the GNSSMeasurement class. As mentioned before, checking the validity of the carrier-phase measurements by using the AccumulatedDeltaRangeState variable is crucial. By looking at the ADR logs from the Xiaomi Mi8 and Samsung S20, it is revealed that these questionable epochs are mainly related to the invalid ADR. However, it is not observed in the Geo++ RINEX file, meaning that it properly handled the invalid ADR by excluding them. In the converted RINEX, such an issue cannot be seen.

Concisely, the results confirmed the importance of evaluating the logging Apps before employing them since these logging Apps are the basics of any smartphone positioning algorithm development. In the next subsection, the UPPP positioning accuracy obtained from the three RINEX files are assessed in the post-processed mode within a kinematic experiment.

### 4.2. UPPP Positioning Accuracy Analysis

**Figure 11:**
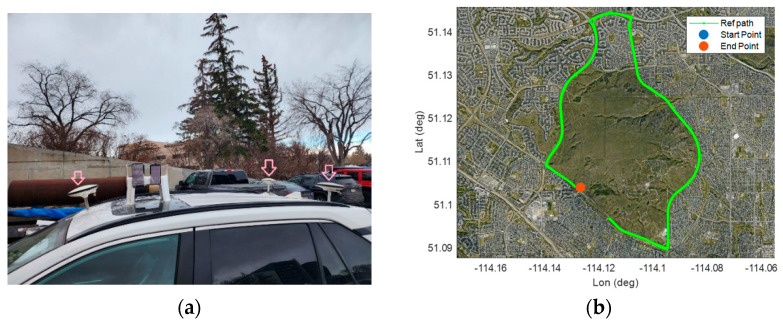
Kinematic experiment done on 22 April 2022 (**a**): test configuration and (**b**): Reference trajectory).

In this subsection, we provide the results of a kinematic test carried out by the same dual-frequency Xiaomi Mi8 device as the static experiment. A kinematic test was carried out on 22 April 2022 with a duration of almost one hour in mostly open-sky environment with overpasses, Calgary, Alberta, Canada. Figure 11 shows the kinematic test configuration and the reference vehicle’s path in this experiment.

The kinematic experiment involved three geodetic receivers (two U-blox F9P and one Septentrio AsteRx-m2), as shown by the three pick arrows in Figure 11, and six smartphones for our future research (here we only used the Xiaomi Mi8 Black dataset). The phones were placed on the vehicle roof. The offsets between all units were measured and applied prior to comparison. The reference trajectory of the vehicle during the kinematic experiment was obtained by the RTK fixed solutions from the three geodetic receivers as the rover receivers. A geodetic receiver on a geodetic pillar (with true position) on the rooftop of the Civil Engineering building, University of Calgary, was also selected as the base receiver. Table 5 provides GNSS data information and processing setting.

**Figure 12:**
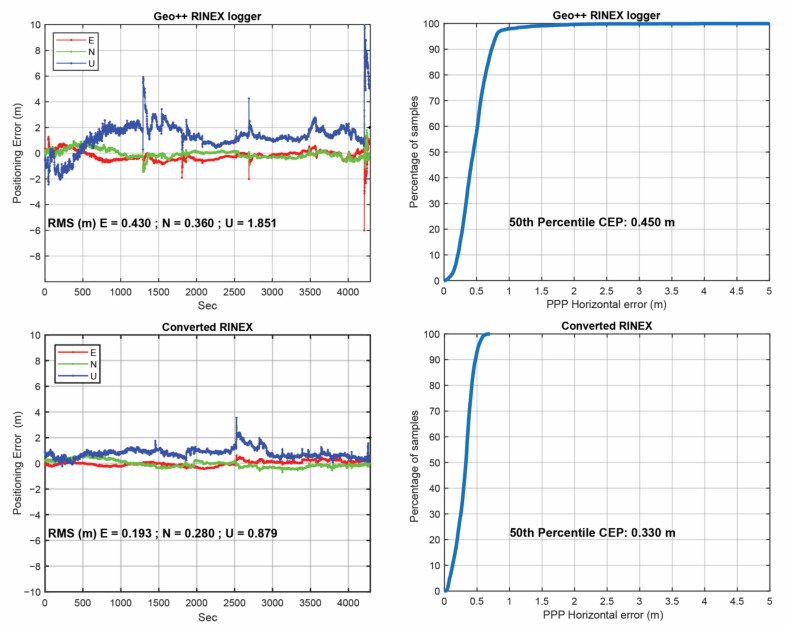
Horizontal positioning errors from Geo++ RINEX logger output and converted RINEX (**left**), cumulative distribution function plot for horizontal positioning error from Geo++ RINEX logger output and converted RINEX (**right**).

Figure 12 provides the positioning errors for Xiaomi Mi8 using the two RINEX files (RINEX by GEO++ RINEX logger and our converted RINEX) in the post-processed mode. The results of the RINEX file from the GnssLogger App were not provided since the obtained accuracy was at the single point positioning (SPP) accuracy level due to the frequent cycle slip detected. It should be noted that the root mean square (RMS) values provided in this figure have been computed using all epochs. In this figure, the cumulative distribution error (CDE) plots for the horizontal positioning error are also provided. The results confirmed the better performance of the converted RINEX in terms of East, North and Up RMS values and the 50th percentile error.

There are many studies devoted to the PPP smartphone positioning, among them we may refer to at least two, [7,43]. In Ref. [7], the Geo++ RINEX logger was used; while in the second study, the authors employed their own developed conversion tool in order to generate the RINEX file [43]. Wu et al. [7] employed the dual-frequency GPS (L1/L5) and Galileo (E1/E5a) observations from a Xiaomi Mi8 smartphone obtained from the Geo++ RINEX logger. Their numerical results showed that the positioning performance of the PPP algorithm employing the ionosphere-free combination was at the meter-level, in kinematic mode. Our positioning accuracy was better than their work, which might be due to differences in measurement environment and employed mathematical model, as well as considering GLONASS observations in our contribution. Chen et al. [43] also proposed a modified single-frequency PPP algorithm in which separate clock biases for pseudorange and carrier-phase observations are estimated. Using a Xiaomi Mi8 smartphone, the average horizontal and vertical RMS error were 0.81 m and 1.65 m, respectively. The difference in accuracy is acceptable since they used the single-frequency data and the predicted IGS products.

Finally, we should mention that, although there are several open-source Apps generating the typical GNSS observations from the Android location API and saving them into the RINEX format, we must still pay more attention to the generation of GNSS observations as we showed some possible issues in the generated observations.

## 5. Conclusions

In this study, we explored the performances of different open-source Apps in generating typical GNSS measurements. We also introduced our newly developed software (namely UofC CSV2RINEX) written in C++ for converting a CSV file into a RINEX file. The quality of raw GNSS observation logged by different smart devices and using different loggers was assessed from different aspects, including the inconsistency between the pseudorange, carrier-phase and Doppler measurements, presence of some carrier-phase observations without changes over time and its possible reasons, etc. Then, the positioning performance of our software was assessed using a kinematic experiment. The conclusions of our study are listed as follows:

1. Consistency between generated pseudorange, carrier-phase and Doppler observations from Android smartphone devices was not fully met in the RINEX outputs of the GnssLogger and Geo++ RINEX Logger Apps. As a highlight, in GnssLogger RINEX file, pseudorange and carrier-phase, observations were not consistent with each other while looking at the CMP combination. In Geo++ RINEX Logger output, the consistency between the carrier-phase and Doppler observations was not met. With our converter software, these three types of measurements were consistent;
2. GnssLogger App had an issue that some carrier-phase observations from the Xiaomi Mi8 and Samsung S20 devices (saved into the RINEX files) did not change over time. These epochs mainly belonged to the lower C/N0 values with invalid ADR states;
3. With our converter software, an improved positioning accuracy could be witnessed when compared with both Geo++ RINEX Logger and GnssLogger outputs. Using UofC CSV2RINEX output, the 50th percentile CEP was 0.330 m, which was 0.450 m for GEO++ RINEX Logger, and SPP-level accuracy for GnssLogger due to frequent cycle slip was detected.

Finally, it should be noted that, to obtain better understanding of the potential reasons of such misbehavior in the typical GNSS observations of the two Apps, a joint effort with their developers is recommended in the future to understand and assess the models and algorithms that have been used to generate the GNSS observations.

## Figures and Tables

**Table 1:** Available GNSS logger Android applications [20].

App	Developer	Output Format
GnssLogger	Google	CSV, NMEA and RINEX
Geo++ RINEX Logger	Geo++ GmbH Company	RINEX
rinexON	FLAMINGO	NMEA, RINEX
GalileoPVT	European Space Agency (ESA)	CSV and NMEA
G-RitZ logger	Ritsumeikan University	NMEA, RINEX
GNSS/IMU Android Logger	Universität der Bundeswehr München	CSV, RINEX and IMU data

**Table 2:** GNSS data information.

Devices	Xiaomi Mi8, Google Pixel 5 and Samsung S20
PRNs	PRN 01 (GPS), 17 (GLONASS), 31 (Galileo)
Mode	Static
App logger	Geo++ RINEX logger (v2.1.6), GnssLogger (v3.0.5.6)
Date	23 November, 2022
Duration	~1 h 30 min
Sampling interval	1 sec

**Table 4:** Consistency between different GNSS observations in three RINEX files.

Combination	GnssLogger	Geo++ RINEX Logger	UofC CSV2RINEX
Code & Phase	**No (Attention required!)**	Yes	Yes
Code & Doppler	No	No	Yes
Phase & Doppler	Yes	**No (Attention required!)**	Yes

**Table 5:** GNSS data information and processing setting.

Device	Xiaomi Mi8
Measurements used	GPS (L1/L5), GLONASS (L1), Galileo (E1/E5a)
Mode	Kinematic
Date	22 November 2022
Duration	1 h
Sampling interval	1 s
Troposphere model	Saastamoinen model
Ionosphere model	Global ionospheric maps (GIM)
Functional model	UPPP model
Stochastic model	C/N0 and elevation weighting function
Elevation mask angle	10 deg
C/N0 mask	20 dB-Hz
Satellite orbit	CODE MGEX precise ephemerides (5 min interval)
Clock error	CODE MGEX precise clock (1 sec interval)
Satellite DCB correction	CAS DCBs in Bias SINEX (BSX) format

## Data Availability

The data used in this study are available on request from the corresponding author.
